# Reversed Effects of Intermittent Theta Burst Stimulation following Motor Training That Vary as a Function of Training-Induced Changes in Corticospinal Excitability

**DOI:** 10.1155/2015/578620

**Published:** 2015-06-17

**Authors:** Tino Stöckel, Jeffery J. Summers, Mark R. Hinder

**Affiliations:** ^1^Human Motor Control Laboratory, School of Medicine, University of Tasmania, Private Bag 30, Hobart, TAS 7001, Australia; ^2^Sport & Exercise Psychology Unit, Department of Sport Science, University of Rostock, Ulmenstraße 69, 18057 Rostock, Germany; ^3^Research Institute for Sports and Exercise Sciences, Faculty of Science, Liverpool John Moores University, Tom Reilly Building, Byrom Street, Liverpool L3 3AF, UK

## Abstract

Intermittent theta burst stimulation (iTBS) has the potential to enhance corticospinal excitability (CSE) and subsequent motor learning. However, the effects of iTBS following motor learning are unknown. The purpose of the present study was to explore the effect of iTBS on CSE and performance following motor learning. Therefore twenty-four healthy participants practiced a ballistic motor task for a total of 150 movements. iTBS was subsequently applied to the trained motor cortex (STIM group) or the vertex (SHAM group). Performance and CSE were assessed before motor learning and before and after iTBS. Training significantly increased performance and CSE in both groups. In STIM group participants, subsequent iTBS significantly reduced motor performance with smaller reductions in CSE. CSE changes as a result of motor learning were negatively correlated with both the CSE changes and performance changes as a result of iTBS. No significant effects of iTBS were found for SHAM group participants. We conclude that iTBS has the potential to degrade prior motor learning as a function of training-induced CSE changes. That means the expected LTP-like effects of iTBS are reversed following motor learning.

## 1. Introduction

Theta burst stimulation (TBS) is a noninvasive brain stimulation (NBS) technique whereby high frequency, subthreshold, bursts of transcranial magnetic stimulation can induce plastic change within human motor cortex (M1). Huang and colleagues [1] reported that intermittent TBS (iTBS) elicited increases in motor evoked potential (MEP) amplitude (indicative of enhanced cortical excitability), whereas continuous TBS (cTBS) resulted in the opposite effects. These and subsequent findings [2–4] are consistent with the view that iTBS and cTBS induce long-term potentiation- (LTP-) like plasticity and long-term depression- (LTD-) like plasticity, respectively.

Metaplasticity describes the manner in which plastic changes within a particular system are affected by that system's recent synaptic history. Homeostatic metaplasticity predicates that a neural system strives to maintain an equilibrium within a particular physiological range, with the threshold for bidirectional plastic change, via long-term potentiation (LTP) or long-term depression (LTD), varying according to its recent synaptic history; for example, prior LTP-like plasticity will raise the threshold for subsequent LTP-like changes while simultaneously reducing the threshold for subsequent LTD-like changes. Understanding the nature of the metaplastic interaction (i.e., homeostatic versus nonhomeostatic) between NBS and motor learning is of particular interest in regard to clinical applications for NBS, where gains in motor performance are the desired behavioral outcome.

In this regard, it has been shown that iTBS has the potential to enhance subsequent motor learning [5] and that cTBS has the potential to degrade subsequent motor learning [6]. These findings reflect the view that motor learning, which itself is thought to be driven by LTP-like changes, is modifiable by inducing prior changes in cortical excitability. Moreover, the findings suggest that the interaction of TBS and motor learning may occur in a nonhomeostatic manner, whereby induction of LTP-like effects via iTBS facilitates, rather than disrupts, the subsequent LTP-like process of motor learning. There is, however, no consensus regarding the behavioral and neurophysiological influence of TBS when administered* following* motor learning. In a single study, Agostino and colleagues [7] reported that iTBS applied to the trained M1 following a short (30 movements, *n* = 17) or a longer (180 movements, *n* = 5) training period did not significantly affect motor training gains. However, no firm conclusions can be drawn from iTBS administered following the short training protocol as little or no learning occurred during training, and the CSE was not modified by the training. While the results showed that iTBS, when applied following the longer motor learning protocol, facilitated CSE without affecting motor performance, this finding was based on data from only 5 participants.

In contrast to the aforementioned studies suggesting* nonhomeostatic* interactions between TBS and subsequent motor learning, studies that employed other NBS techniques, such as paired-associative stimulation (PAS) [8–10] or anodal transcranial direct current stimulation (tDCS) [11, 12], found that the interaction of NBS and motor learning follows the principles of* homeostatic* plasticity. That is, prior motor learning prevented NBS-induced LTP-like plasticity and/or switched LTP-like plasticity to LTD-like plasticity (see [13] for a review).

Accordingly, the present study investigated the effects of iTBS applied to the trained M1 (compared to a sham condition) following a motor training protocol to determine whether iTBS degraded or facilitated the prior motor learning (consistent with homeostatic or nonhomeostatic plasticity) while also assessing associated changes in CSE. Importantly, we extended previous research in this field by complementing group-averages results with individual level analyses to determine the robustness of the findings and to explore associations between neurophysiological and behavioral measures. The findings have important implications for the use of the TBS methods to enhance motor learning, for example, in rehabilitation from traumatic limb injury and stroke.

## 2. Materials and Methods

### 2.1. Participants

Twenty-four right-handed healthy young adults were randomly assigned to either a STIM group (*n* = 12, 5 males, M_age_ = 28.1 ± 6.7 years) or a SHAM group (*n* = 12, 5 males, M_age_ = 24.3 ± 5.1 years). All participants gave written informed consent and completed a medical history questionnaire which confirmed the absence of any known neurological and neuromuscular dysfunction and any contraindications to TMS. All procedures were approved by the Tasmanian Human Research Ethics Committee Network.

### 2.2. Movement Task

Participants performed discrete, isolated, and ballistic abductions of their right index finger (paced at 0.2 Hz) with the goal of maximizing peak horizontal acceleration (see Hinder et al. [14, 15] for further details). A triaxial accelerometer (Dytran Instruments, Chatsworth, CA; Endevco, San Juan Capistrano, CA) was mounted to a plastic splint and taped to the top of the right index finger such that one of the orthogonal axes of the accelerometer was aligned to measure horizontal acceleration. A custom written Signal (CED) script (see [14, 15]) allowed us to detect the first peak of the acceleration trace and provide this as feedback when necessary (see below).

### 2.3. Experimental Procedure

**Figure 1:**
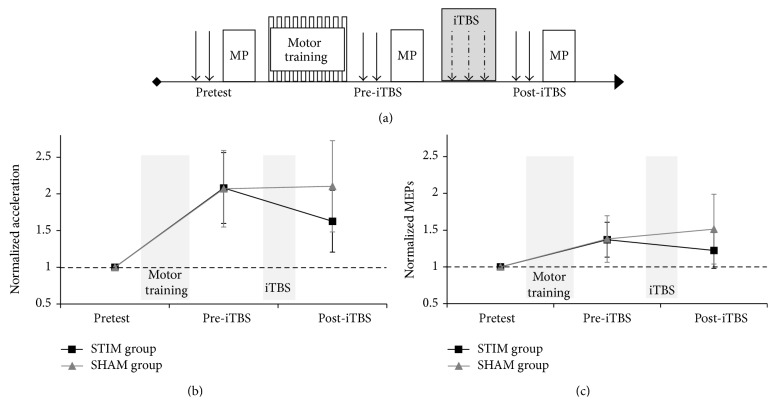
(a) Schematic of the experimental design with assessment of corticospinal excitability (arrows) and motor performance test trials (MP) before motor training (pretest) and before (pre-iTBS) and after iTBS (post-iTBS). Average normalized (b) performance and (c) MEPs of the FDI muscle for the STIM group (black rectangles) and SHAM group (grey triangles). Error bars show 95% confidence intervals (CI) and the horizontal dashed lines represent pretest performance.

Participants practiced the task for 150 movements [14, 15]. Visual feedback was provided on 50% of movements to assist in promoting performance gain. Following training, 600 pulses of iTBS [1] were administered (Magstim Super Rapid^2^ stimulator with 70 mm figure-of-eight-coil) at an intensity of 80% of active motor threshold over the motor hotspot (coil handle 45° to the midline) of the trained first dorsal interosseous (FDI) muscle (STIM group) or over the vertex (handle backwards) with the coil tilted by 90° (coil surface orthogonal to the scalp surface) with one side of the coil remaining in contact with the head [16] (SHAM group). Motor performance (peak acceleration in 10 test movements) and corticospinal excitability, assessed by eliciting MEPs in the trained, right FDI using 15 single pulse MEPs delivered at 130% of resting motor threshold (Magstim 200^2^, 70 mm figure-of-eight-coil; see [14] for more details), were measured before (i.e., pretest) and after motor training (but before iTBS) and again following iTBS. Posttraining (i.e., pre-iTBS) and post-iTBS performance and excitability were normalized to pretest values. CSE testing was conducted in a time window 1.5 to 4 minutes after the cessation of both motor training and iTBS and always preceded motor performance (MP) testing at each of the time points (see Figure 1(a)). Data are reported as mean (normalized) difference (MD) relative to pretest together with corresponding 95% confidence intervals (CI). Partial eta squared and Cohen's *d* are reported as measures of effect size.

## 3. Results

Performance and excitability at pretest following motor training (but prior to iTBS, pre-iTBS) and following iTBS (post-iTBS) are shown in Figure 1 for the STIM group and the SHAM groups.

### 3.1. Motor Performance

Upon completion of the training (i.e., at pre-iTBS), STIM group and SHAM group participants' performance had improved significantly relative to pretest by 108% (MD = 1.08; CI: 0.42, 1.74) and 107% (MD = 1.07; CI = 0.41, 1.73), respectively. Following iTBS (i.e., at post-iTBS), these improvements were reduced to 63% (MD = −0.45; CI: −0.64, −0.26) in the STIM group but remained stable in the SHAM group (MD = 0.04; CI: −0.16, 0.23). A 2 (time: pre-iTBS, post-iTBS) × 2 (group: STIM, SHAM) analysis of variance (ANOVA) revealed a significant main effect of time, *F*(1,22) = 10.37, *p* < 0.01, and *η*_*p*_^2^ = 0.32, indicating a decrease in normalized acceleration across groups following iTBS. This effect was driven by the significant group × time interaction, *F*(1,22) = 14.10, *p* < 0.001, and *η*_*p*_^2^ = 0.39. Sidak adjusted post hoc pairwise comparisons revealed a significant decrease in performance following iTBS in the STIM group (*p* < 0.001, *d* = 0.57), while performance did not change significantly in the SHAM group (*p* = 0.71, *d* = −0.03). Importantly, for the STIM group, analysis at the individual level revealed that all 12 participants exhibited declines in motor performance as a result of the iTBS.

### 3.2. Cortical Excitability

Following motor training (i.e., at pre-iTBS), excitability had increased by 37% (MD = 0.37; CI: 0.01, 0.74) and 38% (MD = 0.38; CI: 0.01, 0.75) relative to pretest levels in STIM and SHAM groups, respectively. At post-iTBS, average normalized MEPs were reduced to 22% above pretest (MD = −0.15; CI: −0.52, 0.22) in the STIM group but were further increased to 51% above pretest (MD = 0.13; CI: −0.24, 0.50) in the SHAM group. Qualitatively, the CSE results mirror those for motor performance; however, a 2 (time: pre-iTBS, post-iTBS) × 2 (group: STIM, SHAM) ANOVA revealed that the main effects and interactions were not statistically significant (all *F* < 1.92, all *p* > 0.18, and *η*_*p*_^2^ < 0.08). At the individual level, MEPs decreased in 8 out of 12 STIM participants as a result of iTBS, while for the majority of the SHAM group MEPs increased or remained relatively unchanged.

### 3.3. Correlations

**Figure 2:**
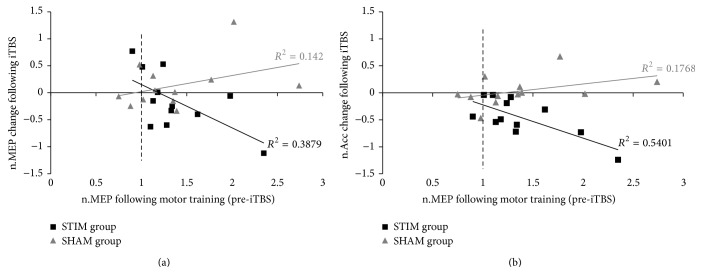
Individual participants' responses to iTBS as a function of normalized MEPs (n.MEP) of the FDI muscle following motor learning (pre-iTBS) for STIM group (black rectangles) and SHAM group (grey triangles). (a) Changes in normalized MEPs following iTBS. (b) Changes in normalized acceleration (n.Acc) following iTBS. Relations between measures are displayed by linear trend lines and *R* ^2^ values for STIM (black) and SHAM group participants (grey). Vertical dashed lines represent MEP values at pretest.

For the STIM group, normalized MEPs following motor learning (at pre-iTBS) were negatively correlated with both the subsequent iTBS-induced change in performance (*r* = −0.73; *p* < 0.01) and the iTBS-induced MEP change (*r* = −0.63, *p* < 0.05) (see Figure 2). However, the iTBS-induced changes in performance and the iTBS-induced MEP change (i.e., post-iTBS relative to pre-iTBS values) were only weakly and not statistically significantly correlated (*r* = 0.40, *p* = 0.20). No significant correlations were found in the SHAM group.

## 4. Discussion

Here we investigated the behavioral and neurophysiological effects of iTBS applied following completion of a 150-movement motor learning paradigm. Motor training resulted in behavioral improvements and corticospinal excitability increases for* all* participants in the cohort. Subsequent administration of iTBS to the trained M1 resulted in statistically significant (group level) declines in motor performance which were evident for* all* individuals together with a concurrent reduction in excitability observed for 8 out of 12 participants (Figure 1). It is clear therefore that the effects of iTBS were remarkably consistent with regard to the behavioral effect (i.e., degradation in motor performance), while the effects with regard to changes in CSE were less robust and somewhat more varied (cf. [4, 17]). Given that all participants in our study exhibited behavioral iTBS-induced effects, it is conceivable that some depotentiation effects may have occurred, whereby iTBS resulted in a change in performance without an overt change in excitability for all participants [11, 18]. However, it may also be the case that the present results represent the growing consensus that a substantial degree of interindividual variability exists with respect to the effects of NBS on CSE. We previously showed that ~2/3 of participants exhibited CSE changes in the “expected” direction following iTBS in two separate sessions [4], with other studies indicating even fewer people responded to iTBS consistent with it inducing LTP-like effects [17]. Similar variability has been observed for cTBS [19] and other protocols such as PAS [20]. Recent evidence suggests that muscle preactivation [21] and factors such as baseline excitability of neural tissue and attention [22] affect responses to NBS. In the current study, at least, an overt change in CSE for each individual was not necessary to result in a behavioral change; rather the behavioral effect of the iTBS was consistent even in the face of some variation in the degree to which iTBS evoked CSE changes.

Importantly, we observed both the extent of the iTBS-induced motor decline and iTBS-induced reduction in excitability varied according to the degree of training-induced increases in excitability (Figure 2); the negative correlation between the use-dependent excitability change and the iTBS-induced plastic change (generally seen as a reduction in excitability) is strong evidence that use-dependent (i.e., learning-induced) LTP-like plasticity and subsequent iTBS-induced plasticity interacted in a* homeostatic* manner. This finding contrasts with studies that have considered the effect of TBS (cTBS and iTBS) on subsequent motor learning [5, 6]; in those studies changes that occurred in motor learning were consistent with a* nonhomeostatic* metaplastic interaction between TBS-induced and motor learning (use-dependent) plasticity. That is, the induction of LTP-like effects via iTBS facilitated, rather than disrupted, the subsequent LTP-like process of motor learning.

Perhaps most noteworthy with regard to the use of iTBS as a potential therapeutic intervention is the strong indication that iTBS induced a LTD-like plastic change following the bout of motor learning rather than the LTP-like changes induced in isolation [1] or prior to motor learning [5]. Crucially, this purported LTD-like effect was consistent across our sample affecting the motor behavior of all participants and reducing the corticospinal excitability in 67% of the cohort, a figure that compares well to the amount of participants responding in the “expected” manner to iTBS when the brain stimulation protocol is conducted in isolation [4, 18].

While this is the first report of a “switch” in the expected plastic change when iTBS is applied to M1 following motor learning, our findings are in line with recent reports on the interaction of motor learning and other NBS techniques, such as PAS [8–10] and anodal tDCS [11, 12]. Taken together, results of the present study and previous work demonstrate that the interaction of NBS and prior motor learning follows the principles of homeostatic plasticity (as discussed by Müller-Dahlhaus and Ziemann [13]). That is, prior motor learning prevents NBS-induced LTP-like plasticity and/or switches LTP-like plasticity to LTD-like plasticity as demonstrated in the present study. A noteworthy finding of the current study was that this switch in the plasticity-inducing effects of iTBS (as evidenced by a significant decline in performance) depends upon the extent of the preceding training-induced excitability changes in M1: performance and cortical excitability decreases following iTBS were largest following larger increases in cortical excitability following motor training. Accordingly, when applying iTBS in therapeutic (i.e., rehabilitation) settings, prior motor learning gains must be taken into account to estimate the efficiency of potential iTBS-induced plastic changes.

## 5. Conclusions

In sum, our results suggest that the expected LTP-like effects of iTBS [1] are reversed following motor learning, which itself is presumed to induce LTP-like plasticity. This effect was particularly robust with regard to its effect on motor performance. The fact that iTBS has the potential to degrade prior motor learning (instead of enhancing it) is of particular interest for the clinical applicability of iTBS. While both motor learning and iTBS separately are purported to have the capacity to support rehabilitation processes in clinical settings, the interaction of both methods seems to depend on factors like prior learning gains or the order of application. As such, future research is necessary to determine the extent to which the current findings of reversed TBS effects hold when TBS is paired with different motor tasks and whether cTBS-induced effects following motor learning are also reversed. Moreover, elucidating more deeply understanding the relationship between iTBS-induced changes in behavior and excitability (which were only weakly associated in this study) is critical for the translation of TBS protocols to clinical (motor rehabilitation) settings.
